# Supplementary material for: Sperm Competition and Paternity in the Endangered Firefly Pyrocoelia pectoralis (Coleoptera: Lampyridae: Lampyrinae)
Source: Insects. 2024 Jan 17;15(1):66. doi: 10.3390/insects15010066 (PMC10817000; doi:10.3390/insects15010066)
Supplement: Supplementary file 1 [file insects-15-00066-s001.zip › insects-2765266-supplementary.pdf]

Supplementary Material

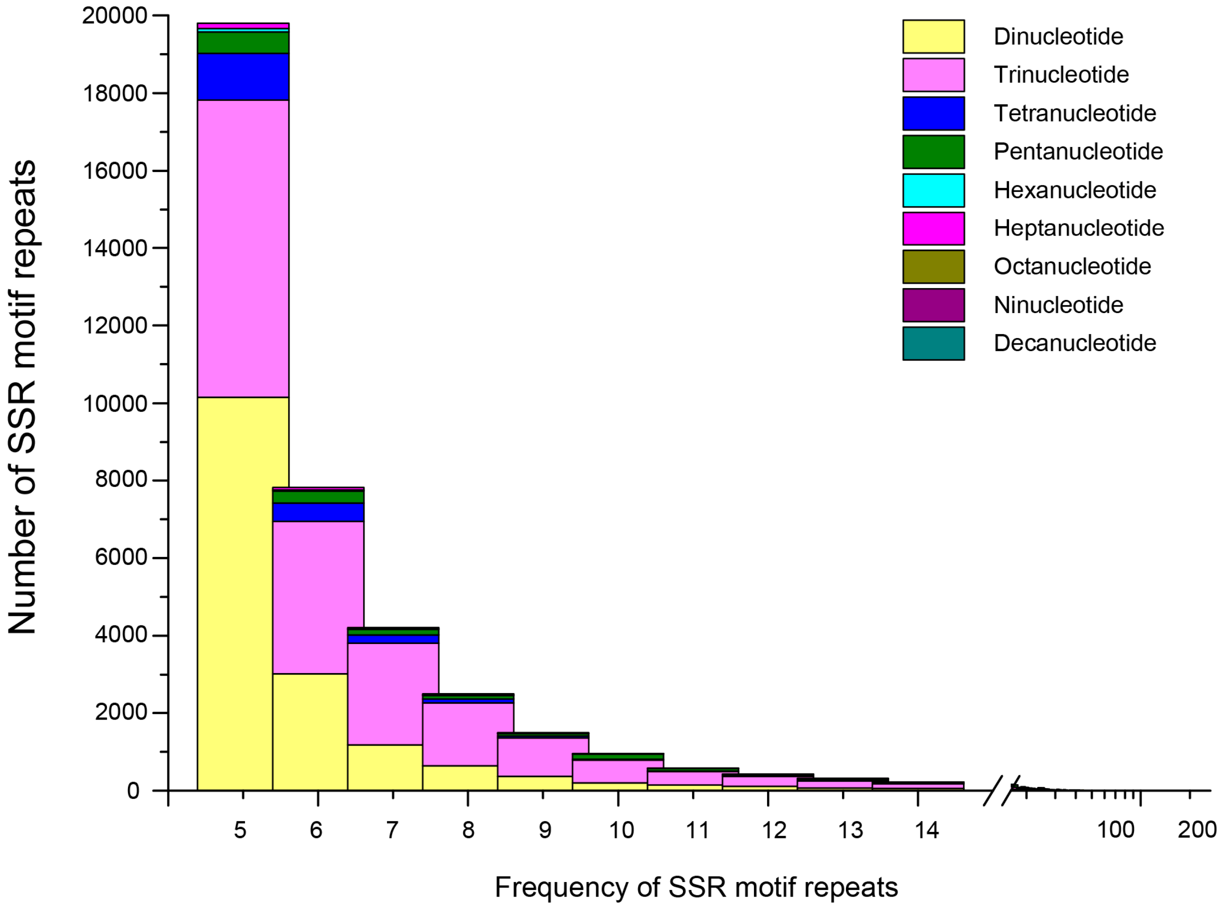

Figure S1 Distribution and frequency of SSR motif repeat numbers. The X-axis shows the frequencies of SSR types, while the Y-axis shows SSR repeat numbers.

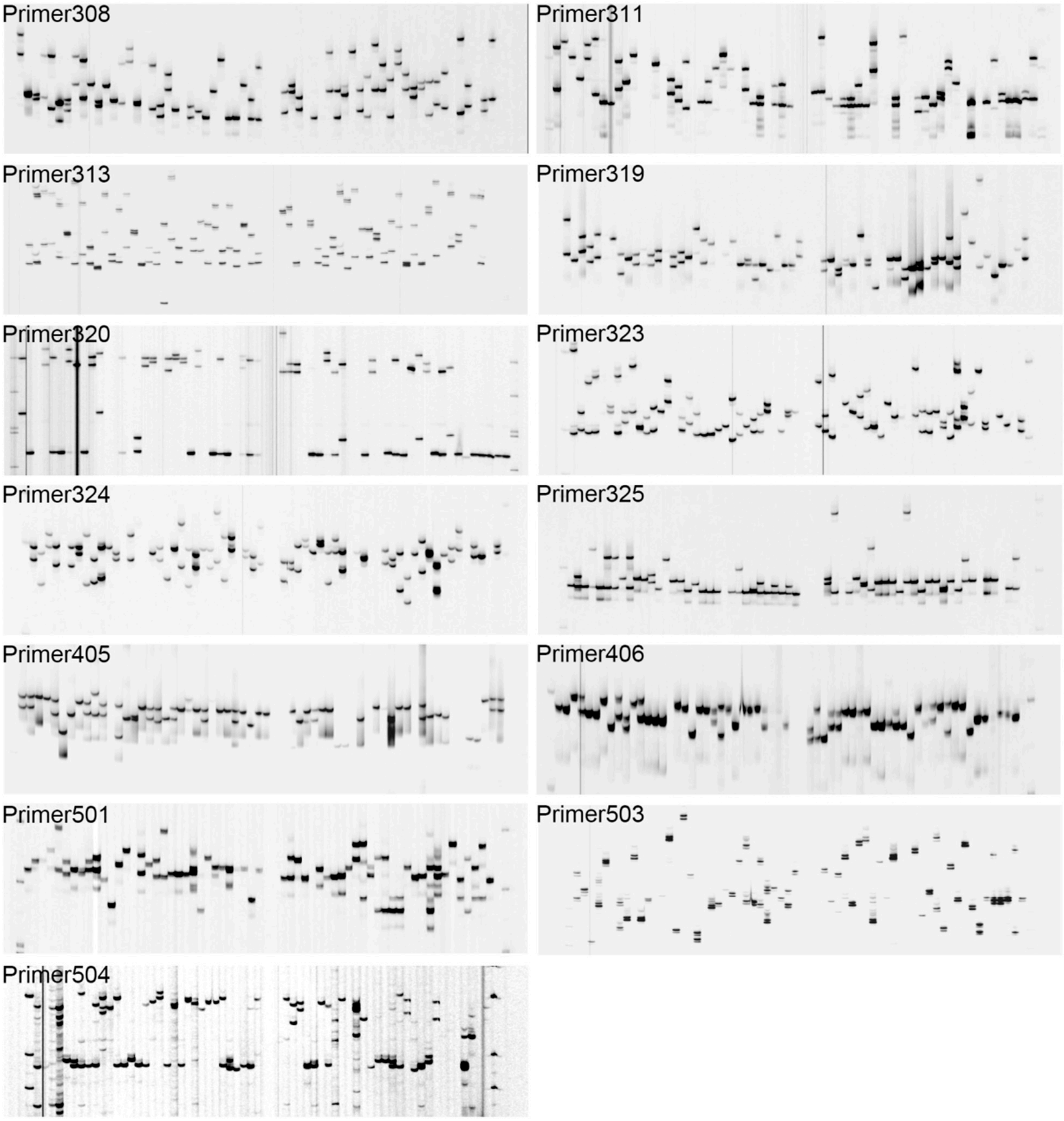

Figure S2 PCR amplification result of 13 SSR loci. 13 pairs of primers showed clear and numerous polymorphism fragments in 6.5 % polyacrylamide gel.

Table S1. SSR markers and characteristics of primers used in genetic analysis of *P. pectoralis*

No	SSR name	Chr	Start	End	Forward primer (5'-3')*	Reverse primer (5'-3')	motif	Result
1	SSR301	LG10	29525644	29524978	TCGGTGTTTCCAGTGTTGT	AACCAGGAGCCAAAGTGT	(gat)6	
2	SSR302	LG07	8220518	8221252	AAAGCAGAAGCCCTACTAAA	AAACGGCCAATACCAGAA	(aat)6	
3	SSR303	LG07	12624283	12624781	CCCTTGGGAAGATTTACAC	AAGACTTGAAGAAAGCTGC	(aat)8	
4	SSR304	LG03	38954751	38955635	AAAAGCTGTGCCTTCTTG	TATTGCTGCTGTTTGGGT	(tta)8	
5	SSR305	LG02	81923098	81922806	ATACATCCATCGCAGAGG	GTGAAGCATTCCCAACAT	(aga)9	
6	SSR306	LG09	13832527	13832008	GCGTGTAAGCATTGTCTC	GCCAAGAATCGGATACATAA	(tac)21	
7	SSR307	LG07	41331038	41330555	CCGCTTACCTCGCTTATT	AAAACATTAGGCGAGGCT	(tat)9	
8	SSR308	LG07	39678327	39677588	CAAAGCGGAACTTCAAAC	AACCAGGTAAAGGTAGCG	(aat)8	OK
9	SSR309	LG06	21371206	21370365	CCGCGCCACTGCTTTAATTT	GAGACGGTCTGTGCTCGATT	(tat)21	
10	SSR310	LG09	27597563	27596993	ATCGTGTTTGTGCCTTTC	GTTTTCACCGCTCGTTCC	(att)13	
11	SSR311	LG04	30577309	30578070	GAACAAATGGTTGAAAGGAA	GCCGGTTGCTATGAGAAT	(taa)12	OK
12	SSR312	LG03	51062472	51062109	GTGAGCGAGTCTCCAGTG	TGTATTCGGTTTGTGCTA	(taa)12	

13	SSR313	LG07	40348582	40349582	ACAAGGCAAACAGAGCAT	CTTCGATCATCTCCGTCA	(gaa)16	OK
14	SSR314	LG06	4767339	4766948	ATTACTTACTCCCGTGTAGAGT	ATGCGGCGTGAAACAACT	(tat)11	
15	SSR315	LG05	47310835	47311093	AGCAATTTCTTGGTGTGGA	ACGACTGACTCGGTCTTAG	(att)16	
16	SSR316	LG07	30337313	30336867	TCACTTTGCTAAGCTGATAAC	TGTGCTAATGCGGACCTA	(aat)13	
17	SSR317	LG02	70162475	70162966	GGAGCAAAACTGGTTCGG	TTATTGCACGACACGGAG	(ttgaa)7	
18	SSR318	LG01	44698570	44697852	TGCCCATTCTTTTTGTCAATGCT	CGCGAGATGTTGAACATGGG	(ata)17	
19	SSR319	LG06	19495493	19494463	ATTTGGGTTAGTTTAGGC	CACGCAAGAAGTGGAGATA	(tat)13	OK
20	SSR320	LG03	48825694	48825192	TAATCCGCGACTGTTTTG	TTGACTCTACCGCACCTC	(ata)12	OK
21	SSR321	LG06	40535170	40536088	TTGACTCTACCGCACCTC	TGGGCGAAAGATGGATGT	(aac)16	
22	SSR322	LG01	63400534	63400170	CAAAGCTGCGTTCATAAT	TTGCCACCATCACAATCT	(tta)14	
23	SSR323	LG08	19419411	19418388	AGGACAGGGCACTCACAT	TGGACATCGCAATCACTA	(ata)15	OK
24	SSR324	LG03	41425698	41426739	TATGTGCTTTGGCTTGTG	AAGTTCGGATGCTAATGC	(tat)12	OK
25	SSR325	LG05	8988867	8989383	CGCTTTTTAGCACCTTCA	ACATACTTCGCCCACTCT	(aat)15	OK
26	SSR401	LG0	24945156	24944269	CTGAGAAACCAGCAT	CGAATCGCATTAAAC	(ttta)8	

		7			CCG	ACATA		
27	SSR402	LG04	21650906	21651620	TGAACCTATGACCCTTGC	TTCGTAATCTTTCACCGC	(tgtc)8	
28	SSR403	LG06	42181662	42181439	TGATTACTTCAATACCCACC	TCCAGATTACATCGCACC	(aatc)6	
29	SSR404	LG03	40217516	40217970	CAGGGATTCTTGAGACACTT	GCTAACTTGACGTAACCCTAAC	(attg)7	
30	SSR405	LG10	10750335	10749891	TTATTTACTGCAAACGGTGAG	GCGAAAGACAGAAAGGGAC	(tatt)6	OK
31	SSR406	LG07	21272843	21273264	TCTTGAATGGACGGACCTA	TTTCTATCCTTGAACGCTGT	(aaat)6	OK
32	SSR501	LG06	43934651	43935197	GCGTGGGCTGAAGAGGAT	TTGTGGAGGTGGCGAAGG	(ttgaa)7	OK
33	SSR502	LG04	40887538	40887995	ACGCACGGGCATTGTCTT	AACCACCCTGGATCACCT	(ttgta)7	
34	SSR503	LG06	7096085	7095375	TGAGCATATATCGAACGTAA	AACAAAGGTGGTTGGTGA	(tacaa)19 (tacag)7	OK
35	SSR504	LG06	12384299	12383838	CTAAAAGTGCTGCTGGCG	TGTTTCAAATACAATGGTCTAA	(atatt)5(att)7	OK
36	SSR505	LG06	11190281	11189675	ATTTCCTCGCACCCTGTA	CCAAAAGGGATGTAAATGAT	(tattg)16	
37	SSR506	LG07	29254037	29255032	CTGACTTATGGCGGCGAA	TGACACCCCAAAAATACTGA	(attac)7	
38	SSR507	LG03	28880322	28880503	AGGCGTGTTACCTTTTCA	GCTTCCCATGCAAACCTT	(taata)6	
39	SSR508	LG0	11190281	11189675	ATTTCCTCGCACCCTG	TCCAAAAGGGATGTA	(tattg)16	

		6			TA	AATGA	
40	SSR509	LG09	9202972	9203267	GTCGCCTGAACAAACACC	TCACATTGGCAAATGACG	(ataac)8
41	SSR510	LG07	29254037	29255032	GACTTATGGCGGCGAACC	ACATAGATCGTAACAAGGCG	(attac)7
42	SSR511	LG07	8254712	8253654	TTCCTCAGGTAAGTCCACCAC	GCCCTATCCACCATTTCG	(tatgg)6
43	SSR512	LG01	2591547	2591933	CGCAAGGGCATTACACGA	TCAGGCGAAAATCAGAAA	(taatt)7
44	SSR513	LG02	19116736	19117247	GCGTCACAATCAACACCT	GGCTTTTACAGAATCTACCG	(ttttg)7
45	IRDye 800-labeled M13f-29 primer				CACGACGTTGTAAAACGAC		

*Each forward SSR primer has a universal M13 19 bp tail sequence added at the 5′ end (5′- CAC GAC GTT GTA AAA CGA C-3′)

Table S2 The body length, width, and weight of mating fireflies (25 female and 59 male).

Firefly	body length (mm)	body width (mm)	weight (g)	Firefly	body length (mm)	body width (mm)	weight (g)	Firefly	body length (mm)	body width (mm)	weight (g)
Female-1	21.23	7.53	0.2740	Male-4	17.10	6.33	0.0822	Male-32	15.33	6.14	0.0631
Female-2	22.11	7.26	0.3132	Male-5	15.74	5.68	0.0711	Male-33	15.26	6.40	0.0622
Female-3	27.08	8.59	0.5890	Male-6	16.68	6.45	0.0663	Male-34	16.88	6.35	0.0678
Female-4	26.49	6.95	0.3303	Male-7	16.32	6.22	0.0698	Male-35	17.27	6.56	0.0631
Female-5	29.21	8.05	0.4718	Male-8	16.41	6.70	0.0611	Male-36	15.76	5.88	0.0468
Female-6	27.76	7.64	0.3482	Male-9	16.59	6.33	0.0647	Male-37	15.67	5.95	0.0450
Female-7	23.45	6.30	0.2992	Male-10	16.44	6.28	0.0764	Male-38	19.23	7.17	0.0946
Female-8	21.37	7.43	0.2780	Male-11	16.94	6.73	0.0829	Male-39	18.67	6.58	0.0679
Female-9	21.51	7.49	0.2785	Male-12	16.69	6.55	0.0964	Male-40	18.86	7.35	0.0832
Female-10	24.03	6.70	0.2884	Male-13	15.10	5.74	0.0699	Male-41	17.96	7.33	0.0836
Female-11	22.00	7.22	0.3032	Male-14	16.86	6.74	0.0739	Male-42	18.38	7.37	0.0793
Female-12	21.44	7.46	0.2782	Male-15	15.34	6.41	0.0532	Male-43	17.66	6.45	0.0833
Female-13	22.00	7.25	0.3092	Male-16	15.35	5.87	0.1121	Male-44	18.70	6.70	0.0685
Female-14	29.10	8.22	0.4705	Male-17	16.88	6.82	0.1028	Male-45	17.54	6.55	0.0617
Female-15	22.83	6.01	0.2693	Male-18	15.93	6.68	0.1112	Male-46	15.05	5.70	0.0444
Female-16	23.03	6.11	0.2993	Male-19	16.69	5.89	0.1231	Male-47	15.88	6.54	0.0575
Female-17	24.04	6.69	0.2783	Male-20	17.04	6.90	0.1013	Male-48	19.39	7.21	0.0944
Female-18	22.00	7.03	0.2650	Male-21	16.81	6.31	0.0919	Male-49	19.03	7.09	0.0744
Female-19	26.42	7.91	0.4464	Male-22	17.54	7.34	0.1022	Male-50	19.49	7.86	0.0977
Female-20	27.00	8.03	0.4760	Male-23	16.77	6.87	0.0978	Male-51	19.27	7.67	0.0968

Female-21	21.59	6.75	0.2633	Male-24	15.95	6.36	0.1003	Male-52	18.66	7.18	0.0833
Female-22	27.00	8.33	0.5877	Male-25	15.80	6.30	0.0643	Male-53	17.93	7.31	0.0841
Female-23	27.03	8.51	0.5891	Male-26	14.32	5.70	0.0635	Male-54	16.34	6.14	0.0623
Female-24	24.04	6.70	0.2777	Male-27	15.45	5.80	0.0667	Male-55	16.48	6.32	0.0654
Female-25	22.00	7.25	0.3092	Male-28	15.74	5.77	0.0622	Male-56	17.89	7.17	0.0610
Male-1	16.24	5.96	0.1524	Male-29	15.42	5.91	0.0656	Male-57	17.70	7.17	0.0767
Male-2	16.38	6.26	0.1400	Male-30	14.68	5.79	0.0637	Male-58	15.92	6.13	0.1121
Male-3	16.13	5.84	0.1424	Male-31	16.28	5.87	0.0666	Male-59	17.14	6.57	0.0690
								Female average	24.23±2.61	7.34±0.70	0.36±0.11
								male average	16.80±1.27	6.50±0.55	0.08±0.02

Table S3 Identification and characteristics of SSR motifs in the *P. pectoralis* genome

Motif	Repeats	Length	Percentage of repeats	Max motif length	Min motif length	Average motif length
Dinucleotide	16370	209166	41.04	538	10	12.76
Trinucleotide	18725	379920	46.94	153	15	20.28
Tetranucleotide	2131	53100	5.34	312	20	24.88
Pentanucleotide	1956	109200	4.9	600	25	55.8
Hexanucleotide	228	12972	0.57	330	30	56.88
Heptanucleotide	297	14091	0.74	294	35	47.39
Octanucleotide	125	6424	0.31	144	40	51.36
Ninucleotide	51	3006	0.13	261	45	58.86
Decanucleotide	7	430	0.02	80	50	61.4
Total	39890	788309	100	600	10	40.8

Table S4 Top15 repeat of different types of SSR motif.

name	Repeats	length	Percentage of repeats
**Total dinucleotide**	**16370**	**209166**	**100%**
TA/AT	6290	80020	38.42%
TG/CA	4977	60186	30.40%
TC/GA	4215	58936	25.75%
GC/CG	888	10024	5.42%
**Total trinucleotide**	**18725**	**379920**	**100%**
ATT/AAT	15578	319896	83.19%
AGA/TCT	1183	24123	6.32%
ACT/AGT	466	9441	2.49%
ATG/CAT	433	7659	2.31%
AGG/CCT	316	6243	1.69%
AAC/GTT	301	5115	1.61%
ACG/CGT	162	2649	0.87%
ACC/GGT	126	2052	0.67%
AGC/GCT	108	1890	0.58%
CCG/CGG	52	852	0.28%
**Total tetranucleotide**	**2131**	**53100**	**100%**
TTTA/TAAA	1190	28912	55.84%

Table S5 Genetic characterization of 13 polymorphic SSR loci of 60 males.

Locus	N	Na	Ne	I	Ho	He	uHe	PIC	HWE
SSR308	60	10	8.706	2.215	0.767	0.885	0.893	0.874	ns
SSR311	60	13	10.811	2.429	0.75	0.908	0.915	0.9	ns
SSR313	60	22	13.74	2.829	0.867	0.927	0.935	0.923	ns
SSR319	60	10	8.582	2.193	0.75	0.883	0.891	0.872	ns
SSR320	60	13	10.169	2.408	0.883	0.902	0.909	0.893	ns
SSR323	60	15	11.392	2.561	0.833	0.912	0.92	0.906	ns
SSR324	60	15	10.762	2.521	0.9	0.907	0.915	0.9	ns
SSR325	60	12	3.169	1.532	0.433	0.684	0.69	0.637	***
SSR405	60	9	7.725	2.111	0.767	0.871	0.878	0.857	ns
SSR406	60	4	2.463	1.042	0.4	0.594	0.599	0.516	***
SSR501	60	9	8.295	2.156	0.733	0.879	0.887	0.867	ns
SSR503	60	34	16.552	3.169	0.483	0.94	0.947	0.937	***
SSR504	60	12	10.959	2.438	0.767	0.909	0.916	0.901	ns
Mean	60	13.692	9.487	2.277	0.718	0.862	0.869	0.845	
SE	0	2.052	1.053	0.148	0.047	0.028	0.029	0.119	

***Highly significant from HWE ($P < 0.001$).

N = Number of examined individuals

Na = No. of different alleles.

Ne = No. of effective alleles = 1/(Sum pi^2).

I = Shannon's Information Index = − 1 * Sum (pi * Ln (pi)).

Ho = observed heterozygosity = no. of Hets/N.

He = expected heterozygosity = 1 − Sum pi^2.

uHe = unbiased expected heterozygosity = (2 N/(2 N − 1)) * He.

PIC = polymorphism information content.

HWE = Hardy–Weinberg equilibrium

ns = no significance

Table S6 Summary of Chi-Square Tests for Hardy-Weinberg Equilibrium (HWE).

Locus	Degrees of freedom	Chi-square value	P-value	Significance
SSR308	45	37.176	0.79	ns
SSR311	78	85.672	0.258	ns
SSR313	231	244.279	0.262	ns
SSR319	45	45.32	0.459	ns
SSR320	78	71.676	0.68	ns
SSR323	105	126.831	0.072	ns
SSR324	105	81.5	0.957	ns
SSR325	66	231.958	0	***
SSR405	36	42.219	0.22	ns
SSR406	6	35.566	0	***
SSR501	36	44.995	0.145	ns
SSR503	561	1088.711	0	***
SSR504	66	82.82	0.079	ns

***Highly significant from Hardy–Weinberg equilibrium ($P < 0.001$)

Ns=no significance

Table S7 Raw data and significance analysis of Paternity assignment

	progeny number allocation			percentage		
Replicates	P1	P2	P3	P1	P2	P3
F1×M2-1	36	15	—	0.7059	0.2941	—
F1×M2-2	20	33	—	0.3774	0.6226	—
F1×M2-3	68	8	—	0.8947	0.1053	—
F1×M2-4	10	40	—	0.2000	0.8000	—
F1×M2-5	2	99	—	0.0198	0.9802	—
F1×M2-6	50	38	—	0.5682	0.4318	—
F1×M2-7	83	48	—	0.6336	0.3664	—
F1×M2-8	55	10	—	0.8462	0.1538	—
F1×M2-9	70	9	—	0.8861	0.1139	—
F1×M2-10	69	6	—	0.9200	0.0800	—
F1×M2-11	21	46	—	0.3134	0.6866	—
F1×M2-12	4	78	—	0.0488	0.9512	—
F1×M2-13	77	23	—	0.7700	0.2300	—
F1×M2-14	9	129	—	0.0652	0.9348	—
F1×M2-15	43	48	—	0.4725	0.5275	—
F1×M2-16	88	11	—	0.8889	0.1111	—
F1×M3-1	19	27	41	0.2184	0.3103	0.4713
F1×M3-2	6	6	81	0.0645	0.0645	0.8710
F1×M3-3	18	0	63	0.2222	0.0000	0.7778
F1×M3-4	0	39	39	0.0000	0.5000	0.5000
F1×M3-5	37	7	14	0.6379	0.1207	0.2414
F1×M3-6	7	5	55	0.1045	0.0746	0.8209
F1×M3-7	4	25	53	0.0488	0.3049	0.6463
F1×M3-8	1	55	5	0.0164	0.9016	0.0820
F1×M3-9	0	0	49	0.0000	0.0000	1.0000

Descriptive Statistics of F1×M2

	N	Mean	SD	SEM
F1×M2-P1	16	0.53816	0.32971	0.08243
F1×M2-P2	16	0.46184	0.32971	0.08243
Difference		0.07633		

t-Test Statistics of F1×M2

	t Statistic	DF	Prob>|t|
Equal Variance Assumed	0.65478	30	0.5176
Equal Variance NOT Assumed	0.65478	30	0.5176

Descriptive Statistics of F1×M3

	Sample Size	Mean	Standard Deviation	SE of Mean
F1×M3-P1	9	0.14586	0.20319	0.06773
F1×M3-P2	9	0.25297	0.29593	0.09864
F1×M3-P3	9	0.60118	0.30403	0.10134

One Way ANOVA of F1×M3

	DF	Sum of Squares	Mean Square	F Value	Prob>F
Model	2	1.02012	0.51006	6.91458	0.00425
Error	24	1.77038	0.07377		
Total	26	2.7905			

Means Comparisons by Fisher Test of F1×M3

		MeanDiff	SEM	t Value	Prob	Alpha	Sig
F1×M3-P2	F1×M3-P1	0.10711	0.12803	0.83658	0.41108	0.05	0
F1×M3-P3	F1×M3-P1	0.45532	0.12803	3.55628	0.0016	0.05	1
F1×M3-P3	F1×M3-P2	0.34821	0.12803	2.7197	0.01195	0.05	1
